# Supplementary material for: Rapid and reversible optogenetic silencing of synaptic transmission by clustering of synaptic vesicles
Source: Nat Commun. 2022 Dec 19;13:7827. doi: 10.1038/s41467-022-35324-z (PMC9763335; doi:10.1038/s41467-022-35324-z)
Supplement: Supplementary file 1 — Supplementary Information [file 41467_2022_35324_MOESM1_ESM.pdf]

Rapid and reversible optogenetic silencing of synaptic transmission by clustering of synaptic vesicles

Dennis Vettkötter[1,2], Martin Schneider[1,2,3], Brady D. Goulden[4], Holger Dill[1,2], Jana Liewald[1,2], Sandra Zeiler[1,2], Julia Guldan[1,5], Yilmaz Arda Ateş[1,5], Shigeki Watanabe[4], Alexander Gottschalk[1,2,*]

1 Buchmann Institute for Molecular Life Sciences, Goethe University; Max-von-Laue-Strasse 15, D-60438 Frankfurt, Germany.
2 Institute of Biophysical Chemistry, Goethe University; Max-von-Laue-Strasse 9, D-60438 Frankfurt, Germany.
3 Max Planck Institute for Neurobiology, Martinsried, Munich, Germany.
4 Department of Cell Biology and Solomon H. Snyder Department of Neuroscience, Johns Hopkins University, 725 N. Wolfe Street, Baltimore, MD 21205, USA.
5 Master Program Interdisciplinary Neurosciences, Department of Biological Sciences, Goethe University Frankfurt, Germany.

* to whom correspondence should be addressed: a.gottschalk@em.uni-frankfurt.de

Supplementary Information

Supplementary Figures

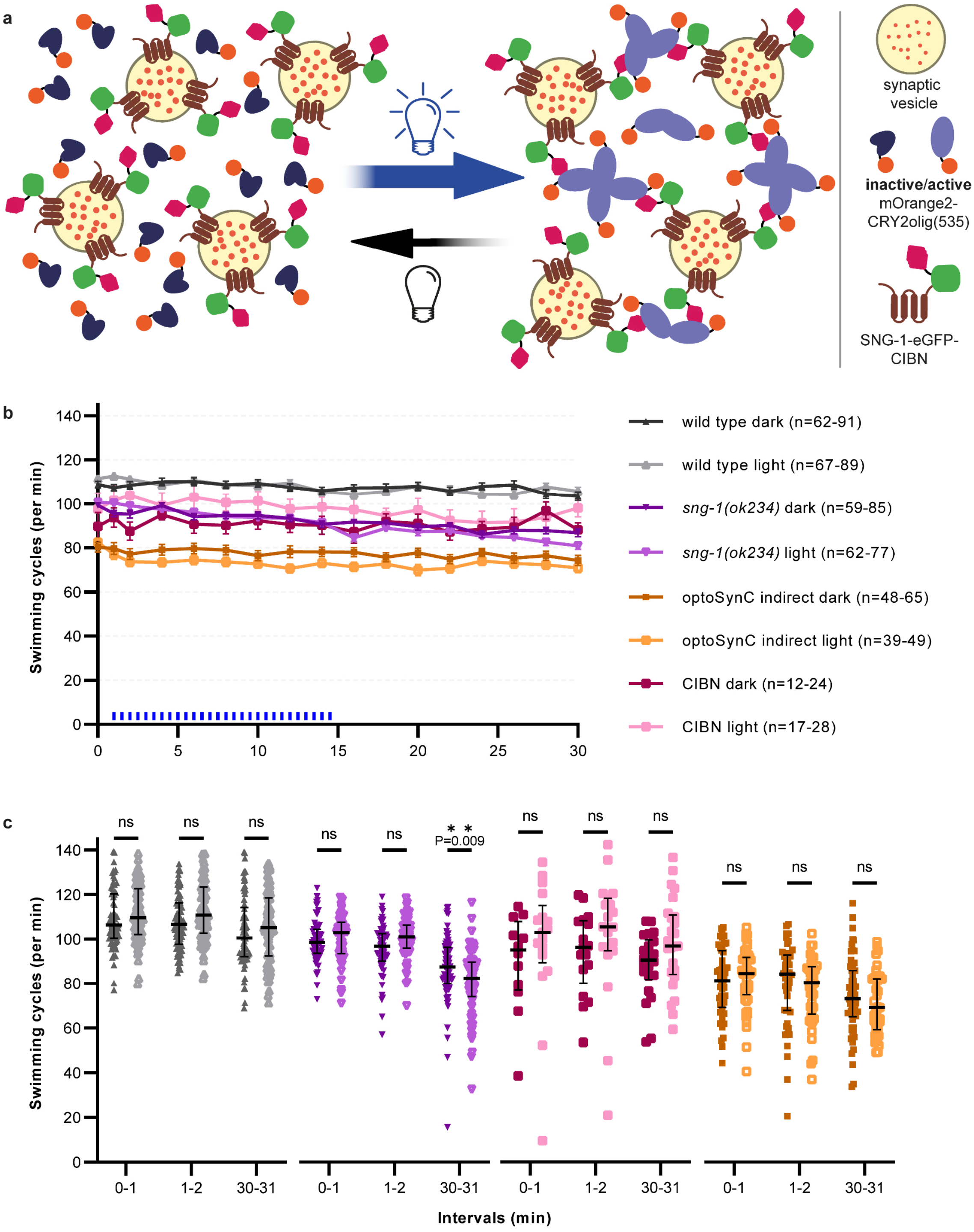

Supplementary Figure 1. A LARIAT-based approach did not affect swimming behavior in *C. elegans*: a Schematic of a panneuronal, LARIAT-based approach ('optoSynC indirect') utilizing heterodimerization of SV membrane bound CIBN and cytosolically expressed CRY2olig(535). **b** Mean ± s.e.m. swimming behavior (cycles) of animals expressing the indicated proteins. Blue light pulses (470 nm, 0.1 mW/mm², 5 s / 25 s ISI) are indicated by blue bars. Number of individual animals (n) across each measured time point and across N independent experiments is indicated. **c** Analysis (median and 25-75 interquartile range) of data in (b), for individual animals, during intervals before (0-1 min), during (1-2 min) and after (30-31 min) blue light illumination. Two-way ANOVA with Bonferroni correction between light and dark; *p<0.05, ns – non-significant. Number of individual animals from left to right: 79, 79, 59, 64, 48, 48, 12, 17, 73, 74, 64, 62, 48, 42, 17, 19, 70, 70, 75, 77, 58, 39, 22, 25. Strains as indicated in b.

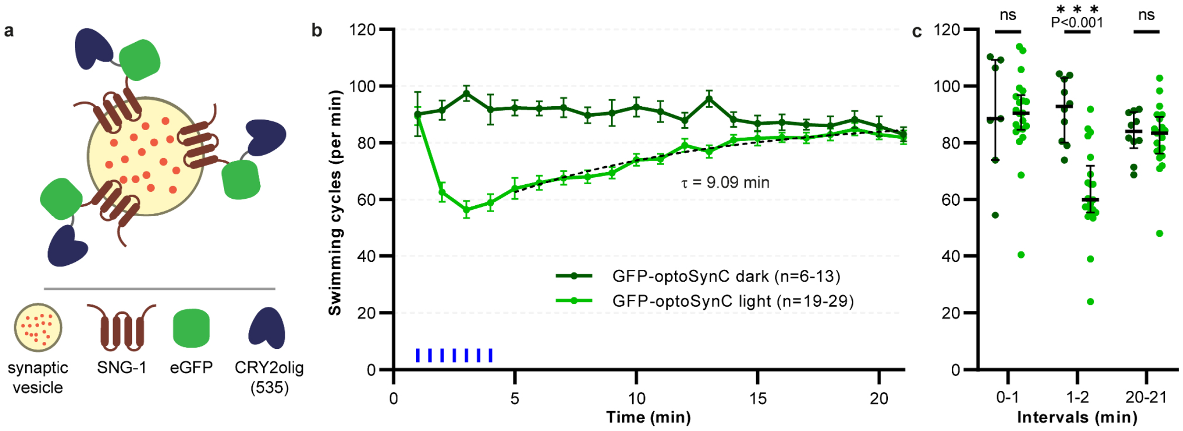

Supplementary Figure 2. Inserting a fluorescent protein in optoSynC reduces its activity: a Schematic illustration of GFP-optoSynC construct. eGFP is inserted between SNG-1 and CRY2olig(535). **b** Mean ± s.e.m. of swimming cycles before, during and after activation of GFP-optoSynC with blue light (470nm, 0.1 mW/mm², 5 s / 25 s ISI, blue rectangles). Dotted line represents one-phase association fit during recovery of swimming behavior. Number of individual animals (n) across each measured time point and over one experiment is indicated. **c** Median and 75-25 IQR of data of individual animals in (b), analyzed in intervals before (0-1 min), during (1-2 min) and after (20-21 min) blue light illumination. Two-way ANOVA with Bonferroni correction; *p<0.05, **p<0.01, ***p<0.001. Number of independent animals from left to right: 7, 22, 10, 21, 10, 21.

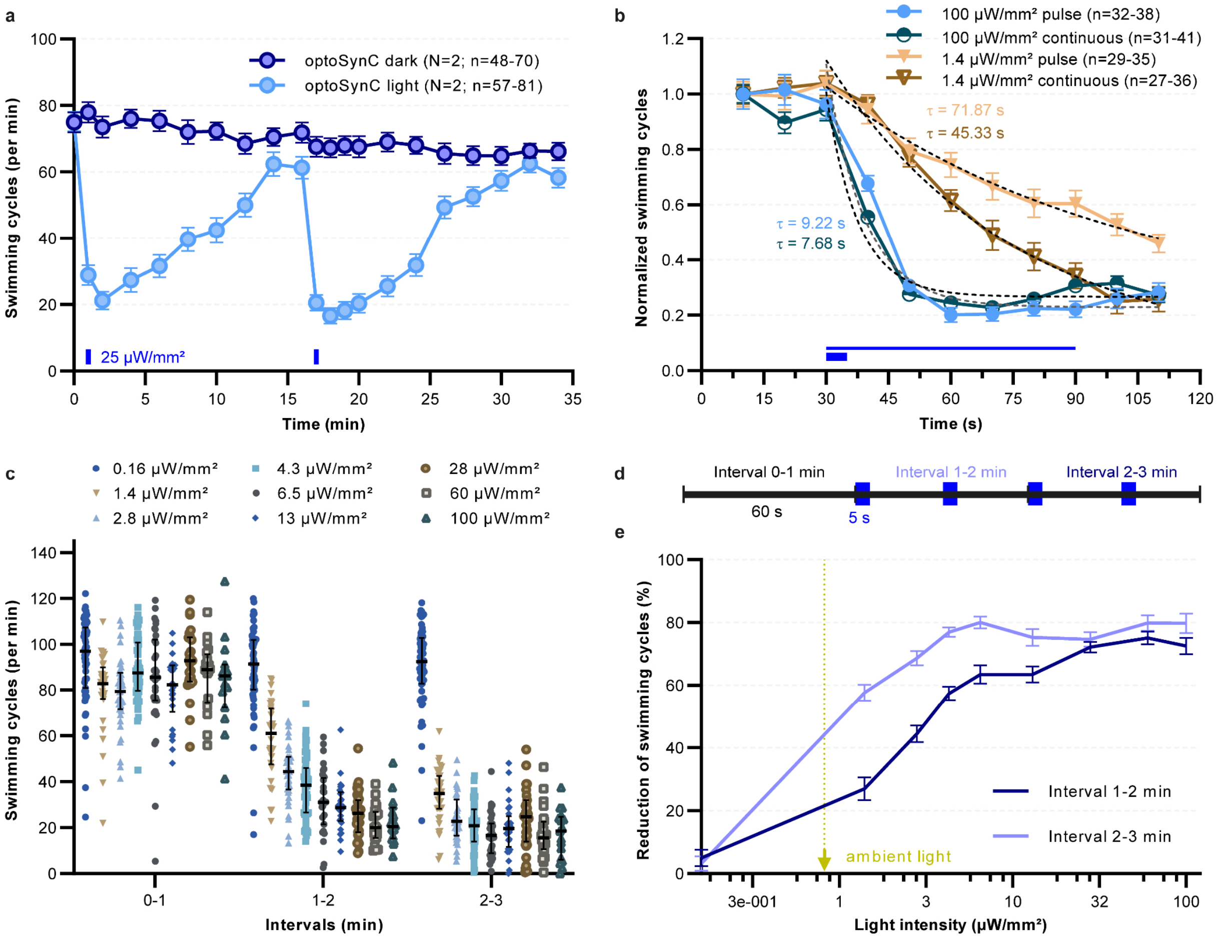

Supplementary Figure 3. Repeated activation, recovery, and parameters required for efficient use of optoSynC (pulse duration, light sensitivity): a optoSynC can be repeatedly activated and recovered. Mean (± s.e.m.) swimming cycles of worms expressing optoSynC with and without blue light illumination. Number of individual animals (n) across each measured time point and across N independent experiments is indicated. **b** OptoSynC is fully activated by a single short pulse of blue light (470 nm, 0.1 mW/mm², 5 s); compare to continuous 1 min illumination. Mean (± s.e.m.) swimming cycles. Number of individual animals (n) across each measured time point and across N independent experiments is indicated. **c** Light sensitivity of optoSynC, tested by swimming behavior of individual animals, as median and IQR; number of animals from one experiment from left to right: 62, 32, 39, 52, 26, 26, 20, 15, 61, 34, 37, 51, 26, 37, 21, 20, 65, 35, 36, 53, 28, 39, 24, 17. **d** Experimental setup for analysis during the indicated intervals. **e** Light titration curve of data from c (mean ± s.e.m.). The indicated intensities of illumination were used during 3 min experiments (see data for individual animals in c and schematic in d. Number of animals as in c.

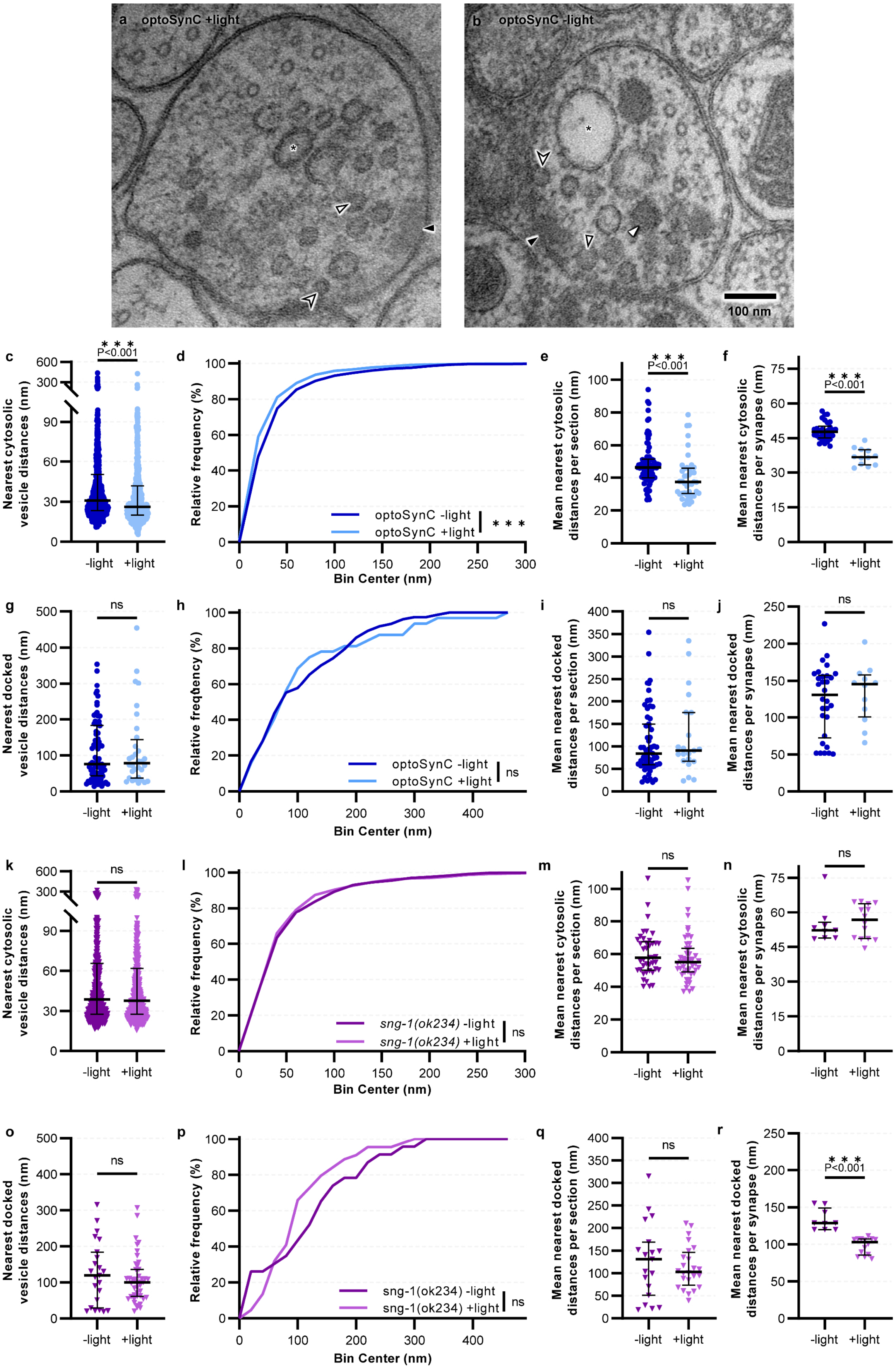

Supplementary Figure 4. Ultrastructural parameters of cholinergic terminals following optoSynC activation: **a, b** Transmission electron micrographs of representative cholinergic synapses from animals illuminated for 5 s (a, +light) with blue light (470 nm, 0.1 mW/mm²) or kept in darkness (b, -light) before high-pressure freezing. Synaptic

vesicles (SVs, open black arrowheads), dense core vesicles (DCVs, white closed arrow heads), dense projection (DP, closed black arrowheads), docked SVs (open black notched arrowhead) and large vesicles (LVs, asterisks) are indicated. Representative example of n=88 (-light) and n=43 micrographs (+light) analyzed. **c** Distance analysis of cytosolic vesicles for each analyzed cholinergic micrograph; -light (n=1327) and +light (n=755). **d** Relative frequency distribution of nearest cytosolic vesicle distances shown in (c). **e** Mean nearest cytosolic distances per section; -light (n=88 sections) and +light (n=43 sections). **f** Mean nearest cytosolic SV distances per synapse; -light (n=30 synapses) and +light (n=12 synapses). **g** Distance analysis of docked vesicles for each analyzed cholinergic micrograph; -light (n=78 docked SVs) and +light (n=32 docked SVs). **h** Relative frequency distribution of nearest docked vesicle distances shown in (g). **i** Mean nearest docked SV distances per section; -light (n=59 sections) and +light (n=21 sections). **j** Mean nearest docked SV distances per synapse; -light (n=30 synapses) and +light (n=12 synapses). **k-r** as for c-j, but analyzing *sng-1(ok234)* controls, without and with illumination prior to high pressure freezing; numbers for docked SVs, sections, and synapses, - light, + light, in k-r: 654, 651(k); 41, 50 (m); 9, 14 (n); 23, 44 (o); 19, 24 (q); 9, 14 (r), respectively. Data shown as median with 75-25% IQR, except in d, h, l, p. Sections originated from two animals; 9-30 synapses for each condition were analyzed. Statistical tests used: Mann-Whitney test (two-tailed) in c, e, g, i-k, m-o, r; Kolmogorov-Smirnov test in d, h, l, p; and unpaired t test (two-tailed) in f, q.; significant differences in (c-n) are given by $^{**}p<0.01$, $^{***}p<0.001$, ns not significant.

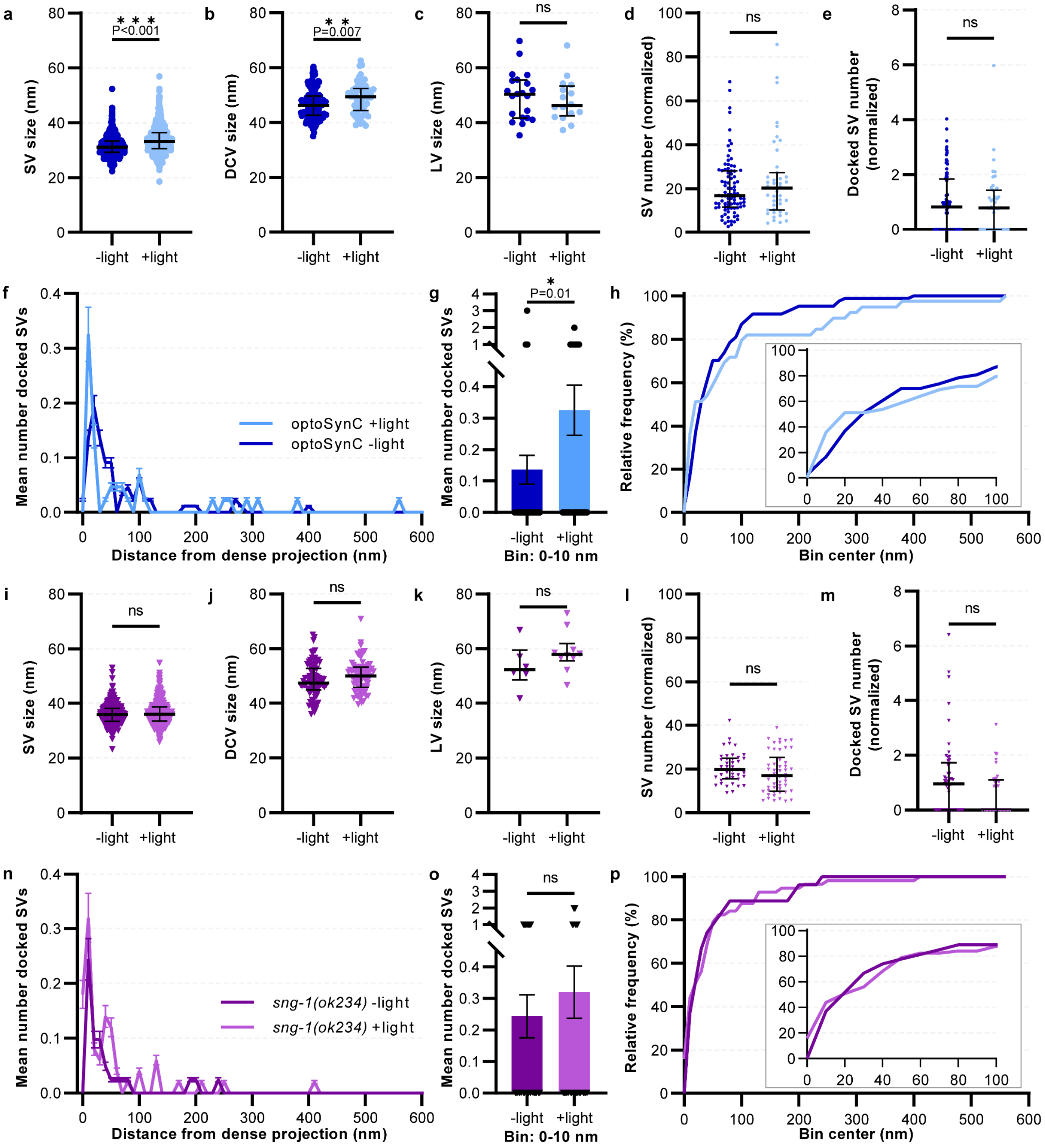

Supplementary Figure 5. Vesicle numbers and distribution in cholinergic terminals following optoSynC activation: a-c Analysis of diameters of SVs (a, n=1561 and 862, from left to right), DCVs (b, n=133 and 56) and LVs (c, n=21 and 16) shown as median with IQR. **d, e** Normalized number of SVs (d) and docked SVs (e) by multiplying with length of PM of each analyzed micrograph and dividing through mean length of PM of each data set (Error bars are s.e.m.; -light (n=88) and +light (n=43)). **f** Distribution of distances of docked SVs relative to the dense projection (DP), along the PM, in 10 nm bins (mean number of docked SVs (± s.e.m.); -light (n=88) and +light (n=43)). **G** Number of docked SVs in bin 1-10 nm, i.e. directly neighboring the DP (mean number of docked SVs (± s.e.m.); -light (n=88) and +light (n=43)). **h** Relative frequency distribution of docked vesicle distances to the DP, shown in (f). Inset shows enlarged region of bins 10-100 nm. **i-p** As for a-h, but showing data for the non-transgenic *sng-1(ok234)* controls. Numbers are (from i-m): 748, 783, 82, 76, 6, 10, 41, 50, 41, 50. For n-o, mean number of docked SVs (± s.e.m.); -light (n=41) and +light (n=50). Statistical tests used were: Mann-Whitney test (two-tailed) in a, b, d, e, i, j, l, m; unpaired t test (two-tailed) in c, g, k, o; and Kolmogorov-Smirnov test in h, p: both ns. Sections originated from two animals, and 9-30 synapses for each condition. Statistically significant differences in (a-e, g, i-m, o) are given by *p<0.01, ***p<0.001, ns not significant.

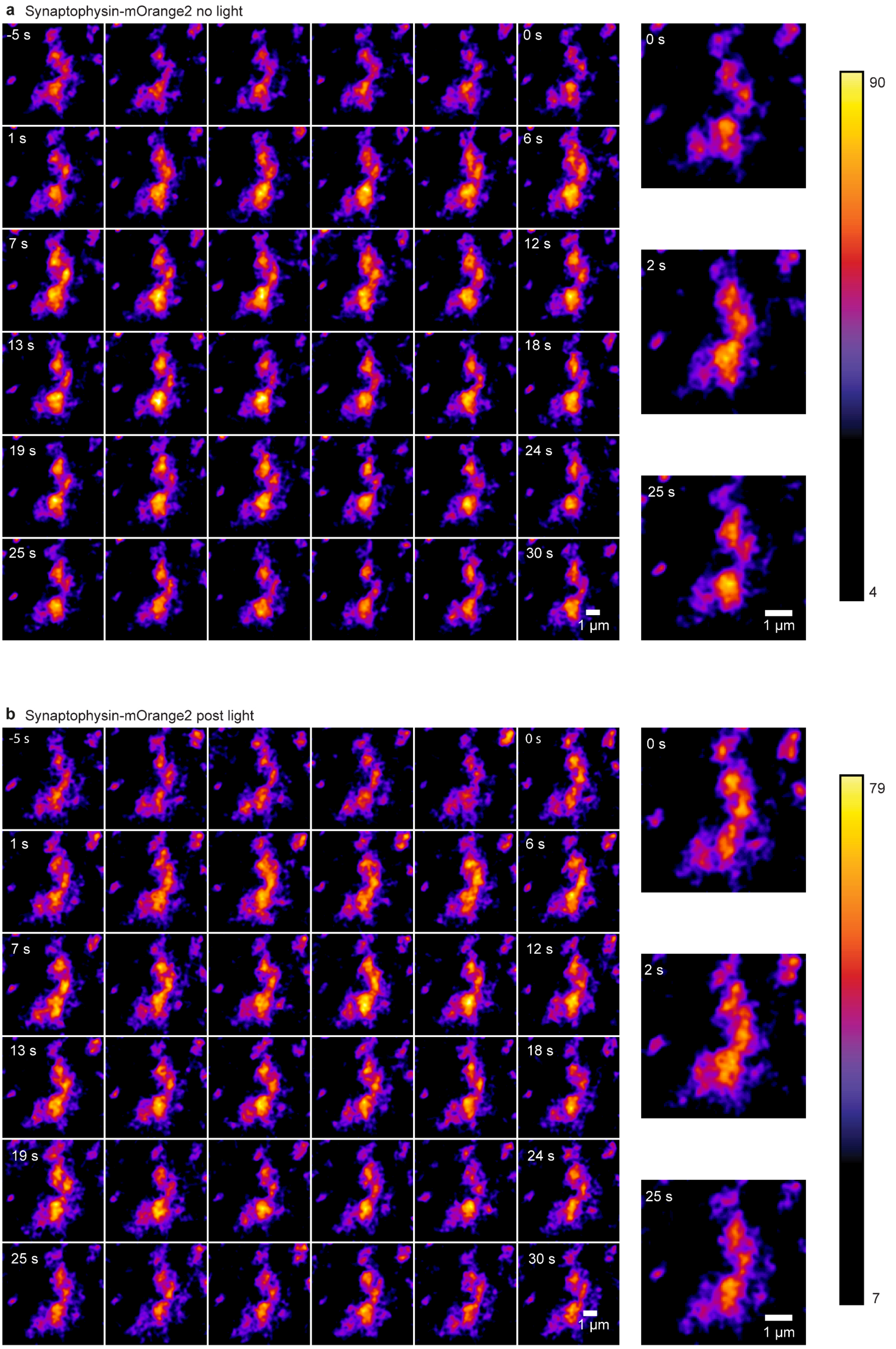

Supplementary Figure 6. Time lapse images of electrically stimulated murine neurons expressing SYP-mOrange, treated with blue light: a Left: Time series of images of hippocampal neuron synapses, imaged relative to the applied electrical field stimulation starting at 0 sec, and without blue light application. On the right, three relevant time points are magnified. Color scale indicates the raw A.U. for fluorescence intensity. **b** As in a, but in neurons that were illuminated with blue light prior to the application of the field stimulation. Scale bars: 1 µm.

a m-optoSynC-mOrange2 no light

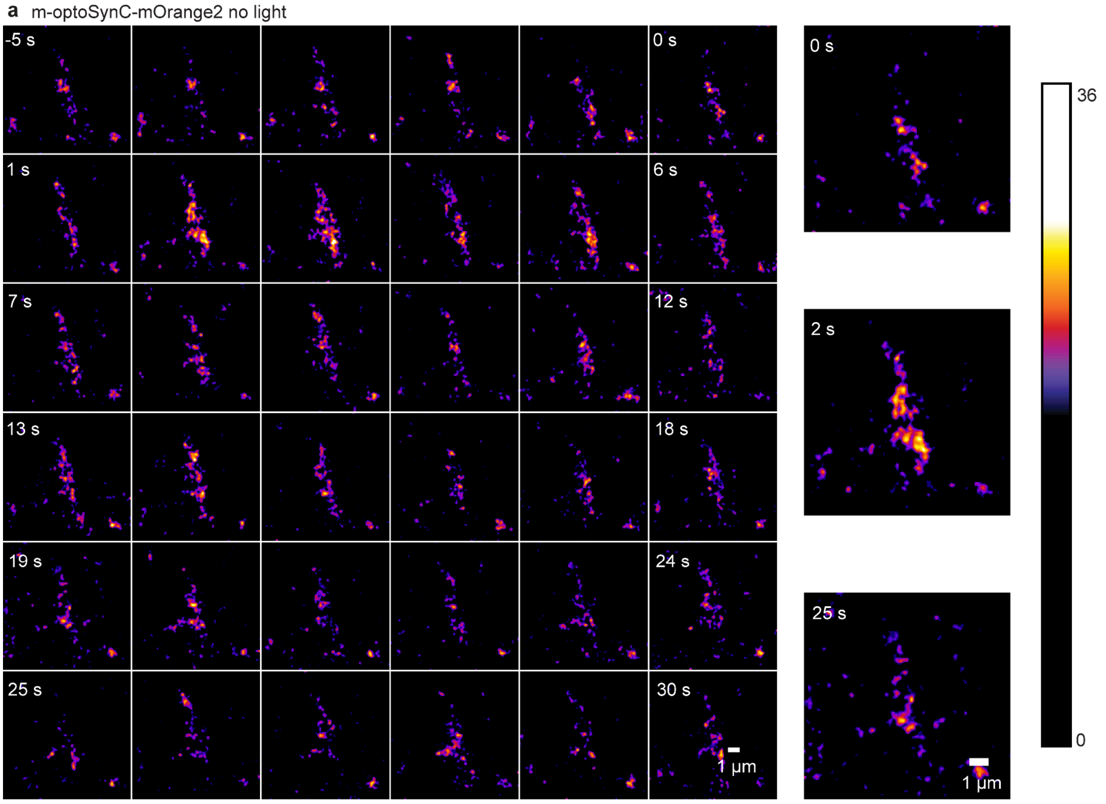

b m-optoSynC-mOrange2 post light

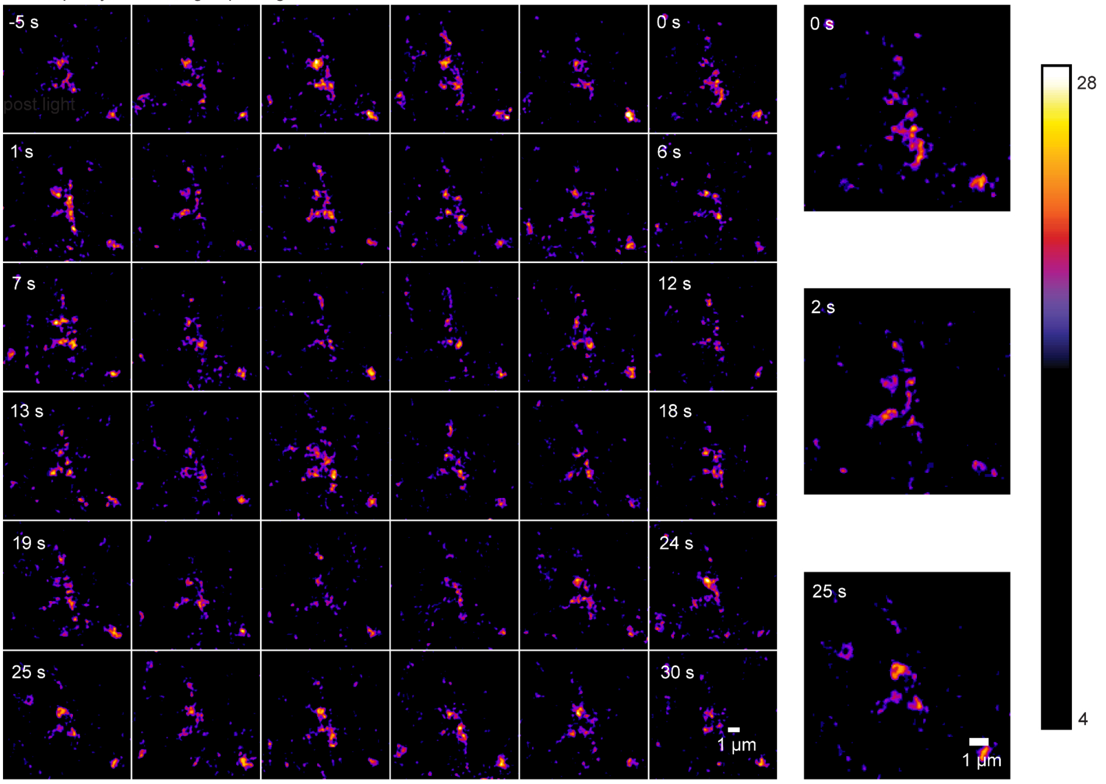

Supplementary Figure 7. Time lapse images of electrically stimulated murine neurons expressing m-optoSynC, treated with blue light: As Supplementary Fig. 6., but in neurons expressing CRY2olig(535) inserted in the SYP-mOrange construct. Color scale represents the raw A.U. for fluorescence intensity.

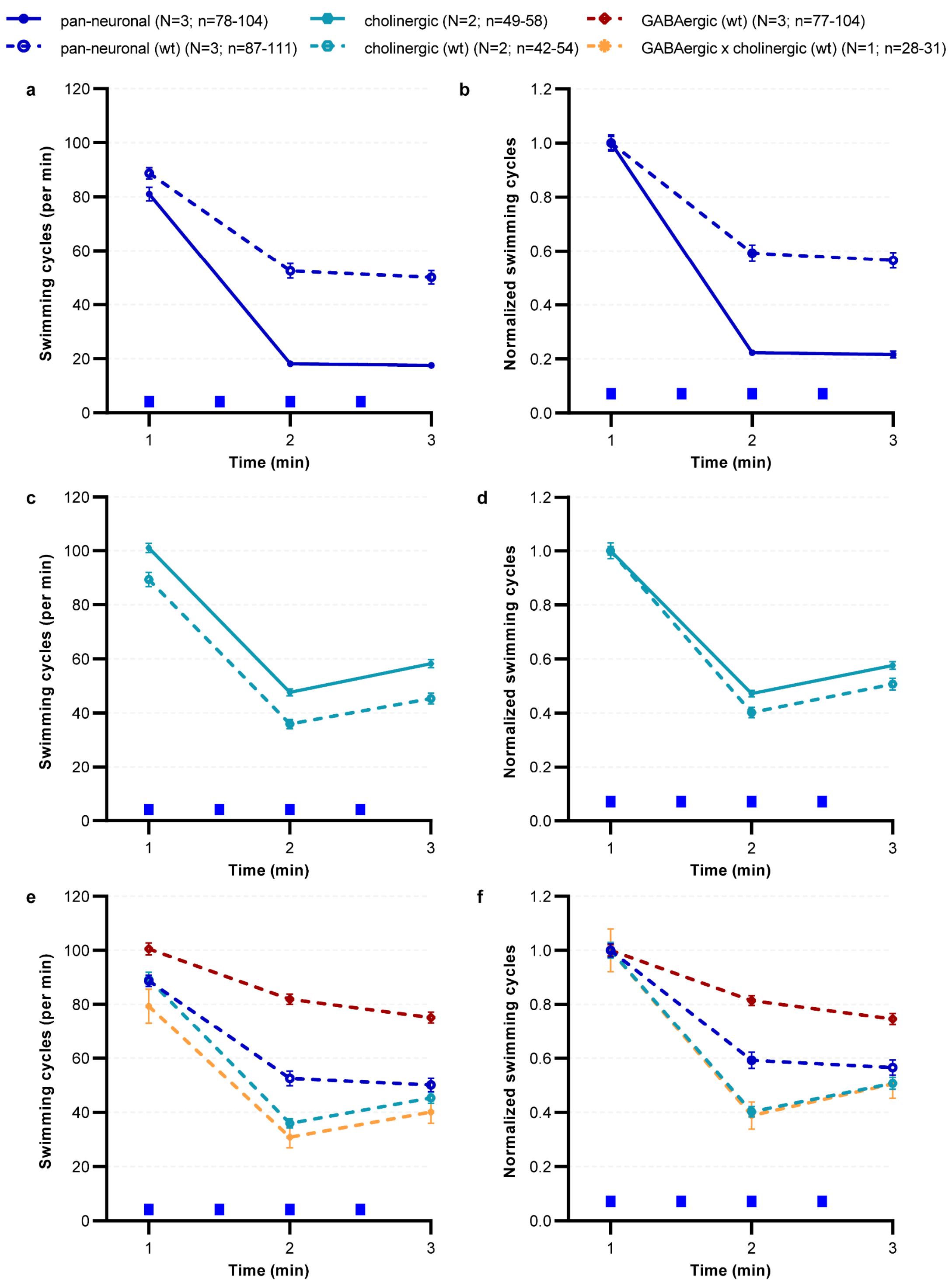

Supplementary Figure 8. Competition between optoSynC and endogenous SNG-1 can reduce optoSynC effects, dependent on neuron type: a, b Mean (± s.e.m.) swimming cycles (normalized in b) before and after activation by blue light (470 nm, 0.1 mW/mm², 5 s / 25 s ISI) was analyzed in animals expressing optoSynC pan-neuronally in wild type background (indicated by dotted line) vs. in *sng-1(ok234)* mutants. **c, d** As in (a, b), but comparing animals expressing optoSynC in cholinergic neurons, in wild type and *sng-1(ok234)* mutants. **e, f** Comparing swimming behavior in animals expressing optoSynC in GABAergic or cholinergic neurons, in both cell types, or pan-neuronally, in wild type background. Number of individual animals (n) across independent experiments (N) is indicated as range.

Supplementary Movies

Supplementary Movie 1: Rapid inhibition of swimming locomotion after optoSynC pan-neuronal photoactivation. Video speed is increased 2x. Blue rectangle indicates application of light pulse. Time is shown as min:sec:centisec.

Supplementary Movie 2: Behavioral response of crawling *C. elegans* before and after optoSynC activation in all neurons. Left: Animals crawling before light stimulus. **Right**, blue border: Animals crawling after light stimulation of optoSynC. Video speed is increased 5x.

Supplementary Movie 3: Behavioral response of zebrafish expressing eGFP pan-neuronally to blue light.

Supplementary Movie 4: Behavioral response of zebrafish expressing synapsin-YFP-CRY2olig(535) (zf-optoSynC) pan-neuronally to blue light.
