# Supplementary material for: Rapid and reversible optogenetic silencing of synaptic transmission by clustering of synaptic vesicles
Source: Nat Commun. 2022 Dec 19;13:7827. doi: 10.1038/s41467-022-35324-z (PMC9763335; doi:10.1038/s41467-022-35324-z)
Supplement: Supplementary file 3 — Description of Additional Supplementary Files [file 41467_2022_35324_MOESM3_ESM.pdf]

Description of Additional Supplementary Files

File Name: Supplementary Movie 1
Description: **Rapid inhibition of swimming locomotion after optoSynC pan-neuronal photoactivation.** Video speed is increased 2x. Blue rectangle indicates application of light pulse. Time is shown as min:sec:centisec.

File Name: Supplementary Movie 2
Description: **Behavioral response of crawling *C. elegans* before and after optoSynC activation in all neurons. Left:** Animals crawling before light stimulus. **Right**, blue border: Animals crawling after light stimulation of optoSynC. Video speed is increased 5x.

File Name: Supplementary Movie 3
Description: **Behavioral response of zebrafish expressing eGFP pan-neuronally to blue light.**

File Name: Supplementary Movie 4
Description: **Behavioral response of zebrafish expressing synapsin-YFP-CRY2olig(535) (zf-optoSynC) pan-neuronally to blue light.**
