# Supplementary material for: Rapid and reversible optogenetic silencing of synaptic transmission by clustering of synaptic vesicles
Source: Nat Commun. 2022 Dec 19;13:7827. doi: 10.1038/s41467-022-35324-z (PMC9763335; doi:10.1038/s41467-022-35324-z)
Supplement: Supplementary file 8 — Reporting Summary [file 41467_2022_35324_MOESM8_ESM.pdf]

nature research

Corresponding author(s): Alexander Gottschalk

Last updated by author(s): 23/11/22

Reporting Summary

Nature Research wishes to improve the reproducibility of the work that we publish. This form provides structure for consistency and transparency in reporting. For further information on Nature Research policies, see our Editorial Policies and the Editorial Policy Checklist.

Statistics

For all statistical analyses, confirm that the following items are present in the figure legend, table legend, main text, or Methods section.

n/a	Confirmed	
☐	☒	The exact sample size (n) for each experimental group/condition, given as a discrete number and unit of measurement
☐	☒	A statement on whether measurements were taken from distinct samples or whether the same sample was measured repeatedly
☐	☒	The statistical test(s) used AND whether they are one- or two-sided<br>*Only common tests should be described solely by name; describe more complex techniques in the Methods section.*
☒	☐	A description of all covariates tested
☐	☒	A description of any assumptions or corrections, such as tests of normality and adjustment for multiple comparisons
☐	☒	A full description of the statistical parameters including central tendency (e.g. means) or other basic estimates (e.g. regression coefficient) AND variation (e.g. standard deviation) or associated estimates of uncertainty (e.g. confidence intervals)
☐	☒	For null hypothesis testing, the test statistic (e.g. F, t, r) with confidence intervals, effect sizes, degrees of freedom and P value noted<br>*Give P values as exact values whenever suitable.*
☒	☐	For Bayesian analysis, information on the choice of priors and Markov chain Monte Carlo settings
☒	☐	For hierarchical and complex designs, identification of the appropriate level for tests and full reporting of outcomes
☒	☐	Estimates of effect sizes (e.g. Cohen's d, Pearson's r), indicating how they were calculated

Our web collection on statistics for biologists contains articles on many of the points above.

Software and code

Policy information about availability of computer code

Data collection	PatchMaster software 2.71 (Heka), MATLAB R2021a (Mathworks), Multi-Worm Tracker 1.3.0, Labview-based custom software (MS-Acqu; hardware-coded).
Data analysis	Data analysis was performed in Origin Pro 2021, Microsoft Excel 2016, 2019; MATLAB R2021a (Mathworks), or ImageJ (1.53c), with statistics calculated in Graph Pad Prism 8.02 and 9. Analysis of mPSCs was done using 'Mini Analysis' software (Synaptosoft, Decatur, GA, USA, version 6.0.7). Multiworm tracking data was analyzed by Choreography (v1.3.0) and the "wrMTrck" plugin for ImageJ. Tracking was validated, non-worm objects manually removed, and data summarized using custom a Python script (https://github.com/dvettkoe/50-percent-tracked). EM data was analyzed using the synapsEM workflow (https://github.com/shigekiwatanabe/SynapsEM). Scripts will be made available upon request.

For manuscripts utilizing custom algorithms or software that are central to the research but not yet described in published literature, software must be made available to editors and reviewers. We strongly encourage code deposition in a community repository (e.g. GitHub). See the Nature Research guidelines for submitting code & software for further information.

Data

Policy information about availability of data

All manuscripts must include a data availability statement. This statement should provide the following information, where applicable:

- Accession codes, unique identifiers, or web links for publicly available datasets
- A list of figures that have associated raw data
- A description of any restrictions on data availability

Data used to generate the analyses and statistics are provided in a supplementary files “raw data and statistics”. Videos from which these data were generated, as well as fluorescence or electron micrographs used, are available from the authors on request, because of large video files.

Field-specific reporting

Please select the one below that is the best fit for your research. If you are not sure, read the appropriate sections before making your selection.

- [x] Life sciences
- [ ] Behavioural & social sciences
- [ ] Ecological, evolutionary & environmental sciences

For a reference copy of the document with all sections, see nature.com/documents/nr-reporting-summary-flat.pdf

Life sciences study design

All studies must disclose on these points even when the disclosure is negative.

Sample size	No statistical methods were applied to predetermine sample size as the effect size was not known before the study. However, sample sizes reported here for the different experiments were matched to published experiments that used similar methodology, model systems and manipulations. For multiworm tracking data, see: Swierczek et al., 2011, doi:10.1038/Nmeth.1625. For EM analysis of C. elegans cholinergic neuron thin sections, see: Schuske et al., 2003, doi: 10.1016/s0896-6273(03)00667-6, or Watanabe et al., 2013, DOI: 10.7554/eLife.00723, or Steuer Costa et al., 2017, http://dx.doi.org/10.1016/j.cub.2016.12.055. For C. elegans electrophysiology data see: Richmond and Jorgensen, 1999, https://doi.org/10.1038/12160, or Bai et al, 2010, DOI 10.1016/j.cell.2010.09.024. For hippocampal neuron mOrange-based SV exo- and endocytosis assays, see: Egashira et al., 2015, DOI:10.1523/JNEUROSCI.4160-14.2015. For zebrafish optogenetics experiments see: Itoh et al., 2014, http://dx.doi.org/10.1016/j.cub.2014.10.065.
Data exclusions	Generally, no data were excluded during the analysis workflow. However, for electron microscopy, we chose synaptic profiles from cholinergic neurons, and discarded such sections that originated most likely from GABAergic neurons, due to their much larger size.
Replication	For experiments involving C. elegans, measurements were performed on at least three independent animal generations and compared to each other (with no significant changes unless noted). These measurements were then pooled for analysis as a single group. The precise n numbers (indicating animals) and N numbers (indicating biological replicates / animals picked from independently grown populations, on different days) are reported in the manuscript.
Randomization	Animals for behavioral analysis were randomly selected. For electrophysiological analysis, there is a bias towards larger animals due to the difficulty of dissection of small animals. Animals for electron microscopy were randomly selected prior high-pressure freezing as well as after the freeze substitution procedure.
Blinding	No blinding was required for C. elegans measurements, since analysis was performed by a consistent workflow, and all single experiments were included in the analyses. Data analysis of behavioral recordings was not done blinded, because optoSynC-mediated effects were apparent and thereby revealed the condition. However, pre-established, semi-automatic stimulation and analysis protocols were used, which could not be influenced by the experimenter, and that did not permit dismissal of any data points or experiments by the analyst based on the analysis result.

Reporting for specific materials, systems and methods

We require information from authors about some types of materials, experimental systems and methods used in many studies. Here, indicate whether each material, system or method listed is relevant to your study. If you are not sure if a list item applies to your research, read the appropriate section before selecting a response.

Materials & experimental systems

n/a	Involved in the study	
x		Antibodies
x		Eukaryotic cell lines
x		Palaeontology and archaeology
	x	Animals and other organisms
x		Human research participants
x		Clinical data
x		Dual use research of concern

Methods

n/a	Involved in the study	
x		ChIP-seq
x		Flow cytometry
x		MRI-based neuroimaging

Animals and other organisms

Policy information about studies involving animals; ARRIVE guidelines recommended for reporting animal research

Laboratory animals	Caenorhabditis elegans strains used:<br>Bristol N2<br>sng-1(ok234)<br>lite-1(ce314)<br>ZX2483: zxEx1146[punc-17::ACR2::eYFP; pmyo-3::QuasAr; pELT::GFP]<br>ZX2577: sng-1(ok234); zxEx1216[psng-1::SNG-1::eGFP::CIBN; pmyo-2::mCherry]

ZX2581: sng-1(ok234); zxEx1224[psng-1::SNG-1::eGFP::CRY2olig(535); pmyo-2::mCherry]
ZX2604: sng-1(ok234); zxIs127[psng-1::SNG-1::CRY2olig(535);pmyo-2::mCherry]
ZX2628: sng-1(ok234); zxEx1234[psng-1::SNG-1::eGFP::CIBN; psng-1::mOrange2::CRY2olig(535); pmyo-2::mCherry]
ZX2737: zxIs132[punc-17::SNG-1::CRY2(535);pmyo-2::mCherry]
ZX2807: zxIs137[ser2prom3::Chrimson::mNeonGreen; pmyo-2::mCherry]
ZX2816: zxEx1277[punc-47::SNG-1::CRY2olig(535); pmyo-3::mCherry]
ZX2865: zxIs137; zxEx1291 [ser2prom3::SNG-1::CRY2olig(535); pmyo-3::mCherry]
ZX2871: zxIs132; zxEx1277[punc-47::SNG-1::CRY2olig(535); pmyo-3::mCherry]
ZX2872: lite-1(ce314); zxIs127[psng-1::SNG-1::CRY2olig(535);pmyo-2::mCherry]
ZX2911: zxIs127[psng-1::SNG-1::CRY2olig(535);pmyo-2::mCherry]
ZX2914: sng-1(ok234); zxIs132, ZX2950: sng-1(ok234); zxEx1322[psng-1::SNG-1::CRY2(D387A)olig(535)::SL2::mCherry; pmyo-2::CFP]

For Zebrafish (Danio rerio) experiments, embryos of one, three and four days of age were used, as described in the materials and methods section. Sex of individual animals can not be determined at these developmental stages. Embryos were obtained by mating adult female with adult male zebrafish of strain Tg(elavl3.2:Gal4-VP16)mde4 (strain #20433, European Zebrafish Resource Center). Adult zebrafish were maintained at 28 °C, constant water supply and a 14/10 h light/dark cycle in accordance with the respective FELASA guidelines and the European Directive 2010/63/EU. Experiments involving genetically modified animals were done with acutely injected embryos (no transgenic lines were generated), and no experiments were done including animals > 4 days of age, thus not requiring official approval.

For experiments with murine neurons, hippocampal cultures were prepared from C57/BL6-N mouse embryos (day 18) of both sexes. Mice were maintained in accordance with the Johns Hopkins Animal Care and Use Committee regulations. These include ad libitum access to food and water, sterile environment with enrichment items, weekly cage changes, temperature control at 22°C, and a 12 hour light / 12 hour dark cycle. Once hippocampal cultures were expressing m-optoSync (following lentiviral infection), cultures were kept in a humidity-controlled incubator at 37 C, with 5% CO_2, in low light environments (lights off, with small amounts of ambient light) when changing media and setting up imaging experiments.

Wild animals	This study did not involve wild animals.
Field-collected samples	This study did not involve samples collected from the field.
Ethics oversight	Studies on C. elegans do not require ethics oversight.<br>For zebrafish: All experiments employing zebrafish were conducted according to the European Directive 2010/63/EU on the protection of animals used for scientific purposes and the animal research board of the State of Hessen.<br>For mice: All the mouse experiments were performed in accordance with rules and regulations of the National Institute of Health, USA, and animal protocols were approved by committee of animal care, use of the Johns Hopkins University.

Note that full information on the approval of the study protocol must also be provided in the manuscript.
